# Anti-Inflammatory Effect of Rhapontici Radix Ethanol Extract via Inhibition of NF-*κ*B and MAPK and Induction of HO-1 in Macrophages

**DOI:** 10.1155/2016/7216912

**Published:** 2016-07-25

**Authors:** Yun Hee Jeong, You-Chang Oh, Won-Kyung Cho, Nam-Hui Yim, Jin Yeul Ma

**Affiliations:** Korean Medicine (KM) Application Center, Korea Institute of Oriental Medicine, 70 Cheomdan-ro, Dong-gu, Daegu 41062, Republic of Korea

## Abstract

Rhapontici Radix (RR) has been used in traditional medicine in East Asia and has been shown to have various beneficial effects. However, its biological properties or mechanism on inflammation-related diseases is unknown. The goal of this study was to determine the anti-inflammatory activity and underlying molecular mechanisms of Rhapontici Radix ethanol extract (RRE). The inhibitory effect of RRE on the production of NO, cytokines, inflammatory-related proteins, and mRNAs in LPS-stimulated macrophages was determined by the Griess assay, ELISA, Western blot analysis, and real-time RT-PCR, respectively. Our results indicate that treatment with RRE significantly inhibited the secretion of NO and inflammatory cytokines in RAW 264.7 cells and mouse peritoneal macrophages without cytotoxicity. We also found that RRE strongly suppressed the expression of iNOS and COX-2 and induced HO-1 expression. It also prevented nuclear translocation of NF-*κ*B by inhibiting the phosphorylation and degradation of I*κ*B*α*. Furthermore, the phosphorylation of MAPKs in LPS-stimulated RAW 264.7 cells was significantly inhibited by RRE. These findings suggest that RRE may operate as an effective anti-inflammatory agent by inhibiting the activation of NF-*κ*B and MAPK signaling pathways and inducing HO-1 expression in macrophages. Our results suggest that RRE has potential value as candidate to inflammatory therapeutic phytomedicine.

## 1. Introduction

Inflammation is a highly regulated defensive process that results from tissue injury or infection. However, excessive inflammation can cause severe chronic diseases, such as asthma, arthritis, vascular diseases, and cancer [1]. Macrophages play a key role in the regulation of inflammatory responses initiated in response to an infection or accumulating damaged or dead cells. Lipopolysaccharide (LPS) treatment of the murine macrophage cell line RAW 264.7 results in the production of many inflammatory mediators, such as nitric oxide (NO), inducible nitric oxide synthase (iNOS), cyclooxygenase- (COX-) 2, tumor necrosis factor- (TNF-) *α*, interleukin- (IL-) 6, and IL-1*β* [2–4]. The expression of these factors is induced by the activation of nuclear factor- (NF-) *κ*B and mitogen-activated protein kinases (MAPKs) in macrophages [5–7]. NF-*κ*B and MAPK pathways, two important inflammatory signaling pathways in macrophages, induce the expression and release of a wide range of inflammatory mediators that orchestrate the host's inflammatory responses. A number of previous studies have shown that various naturally derived substances exert anti-inflammatory effects by inhibiting the NF-*κ*B and MAPK signaling pathways [8–10].

NF-*κ*B is a ubiquitous transcription factor that activates many inflammatory genes. There are five proteins in the mammalian NF-*κ*B family, including NF-*κ*B1 (p105/p50), NF-*κ*B2 (p100/p52), p65 (Rel A), c-Rel, and RelB [11, 12]. Among them, the p65/p50 heterodimer is important in transcriptional activation. In unstimulated cells, the inhibitory protein I*κ*B sequesters NF-*κ*B p50/p65 heterodimers in the cytoplasm. Upon stimulation by inflammatory stimulants, such as LPS, the I*κ*B kinase complex is phosphorylated and degraded. Once released, NF-*κ*B translocates into the nucleus to increase the expression of various cytokines, including TNF-*α*, IL-6, and IL-1*β*. NF-*κ*B also activates inflammatory pathways involving iNOS and COX-2 [13–15]. Given these functions, it has been suggested that inhibitors of NF-*κ*B function may be useful as anti-inflammatory agents. The MAPK family, including extracellular regulated kinases (ERK), c-Jun N-terminal kinases (JNK), and p38, also induces the production of inflammatory molecules and immune-related cytotoxic factors [16, 17]. Several studies have also demonstrated that MAPKs play a significant role in the activation of NF-*κ*B [18, 19]. Therefore, it may potentially be advantageous to treat inflammatory diseases by suppressing the activation of NF-*κ*B and MAPK [20].

NO is generated by NO synthases (NOS), which regulate the expression of proinflammatory mediators and inflammatory cytokines important in inflammation and the immune response. The expression of iNOS is also closely linked with the induction of heme oxygenase- (HO-) 1, which plays an important role in inflammation [21]. In inflammatory conditions, HO-1 reduces the expression of proinflammatory mediators, such as NO, TNF-*α*, IL-6, and IL-1*β*. A number of therapeutic agents exert their anti-inflammatory effects by increasing HO-1 expression.

Rhapontici Radix (RR) is the dried root of* Rhaponticum uniflorum* (L.) DC, called “Nuro” in Korea. RR is widespread throughout Asian countries but is primarily produced in Korea and China. Chemical components of RR mainly include ecdysterone, flavonoids, triterpenoid saponins, volatile oil, and organic acids. RR has been traditionally used as an herbal medicine to treat inflammatory diseases in Korea, but its anti-inflammatory activities and detailed mechanism of action remain to be understood.

Therefore, the objective of this study is to elucidate the modulating effects of Rhapontici Radix ethanol extract (RRE) on the production of NO, TNF-*α*, IL-6, IL-1*β*, iNOS, and COX-2 via blockade of NF-*κ*B and MAPK signaling pathways or induction of HO-1 in RAW 264.7 cells and mouse primary macrophages. Also, this experiment is carried out in order to elucidate the potential anti-inflammatory effects and mechanisms of RRE within pharmacologically noncytotoxic concentration levels.

## 2. Materials and Methods

### 2.1. Preparation of RRE

*Rhaponticum uniflorum* belongs to the Compositae family. RR was purchased as a dried herb from Yeongcheonhyundai Herbal Market (Yeongcheon, Korea) and was identified by Professor KiHwan Bae, Chungnam National University, Korea. All voucher specimens were deposited in an herbal bank at the KM-Application Center, Korea Institute of Oriental Medicine (voucher number: E212). The dried herb (30.0 g) was extracted with 390 mL 70% ethanol in a 37°C shaking incubator (100 rpm) for 24 h. The extract solution was filtered using 185 mm filter paper (Whatman, Piscataway, NJ, USA) and concentrated using a rotary vacuum evaporator (Buchi, Tokyo, Japan). Samples were freeze-dried and stored in a desiccator at 4°C before use. The sample acquisition was 1.3957 g and the yield was 4.6523%.

### 2.2. Reagents and Cell Culture

Murine macrophage-like RAW 264.7 cells were obtained from American Type Culture Collection (ATCC, Manassas, VA, USA). Roswell Park Memorial Institute (RPMI) 1640 medium, antibiotics, and fetal bovine serum (FBS) were purchased from Lonza (Basel, Switzerland). LPS and bovine serum albumin (BSA) were obtained from Sigma (St. Louis, MO, USA). A cell counting kit (CCK) and enzyme-linked immunosorbent assay (ELISA) antibody sets were purchased from Dojindo Molecular Technologies, Inc. (Kumamoto, Japan), and eBioscience (San Diego, CA, USA), respectively. Primary antibodies for iNOS, COX-2, HO-1, phospho-ERK, ERK, phospho-p38, p38, phospho-JNK, JNK, phospho-I*κ*B*α*, I*κ*B*α*, NF-*κ*B p65, TATA box-binding protein (TBP), and horseradish peroxidase-conjugated secondary antibodies were acquired from Cell Signaling Technology, Inc. (Boston, MA, USA). *β*-actin antibody was purchased from Santa Cruz Biotechnology (Santa Cruz, CA, USA). Nitrocellulose membrane was obtained from Millipore (Bedford, MA, USA). RNA extraction and DNA synthesizing kits were purchased from iNtRON (Seongnam, Korea) and Bioneer (Daejeon, Korea), respectively. RAW 264.7 cells were grown in complete RPMI 1640 medium supplemented with penicillin (100 U/mL), streptomycin (100 *μ*g/mL), and 10% heat-inactivated FBS at 37°C in a humidified incubator with 5% CO_2_.

### 2.3. Preparation of Mouse Peritoneal Macrophages

Male BALB/c mice (25 ± 3 g) were purchased from Samtako BioKorea (Osan, Korea). To obtain mouse peritoneal macrophages, mice were treated with 300 *μ*L of sterile 3% sodium thioglycollate (Sigma, St. Louis, MO, USA) 3 days before they were sacrificed by cervical amputation. Animals were housed at five per cage in an air-conditioned room at 22–24°C with a 12 h dark/light cycle and were given food and water* ad libitum*. Peritoneal macrophages were harvested by washing their peritoneal cavity with 10 mL of ice-cold phosphate-buffered saline (PBS) and massaging the abdomen gently for 3 min. The peritoneal fluid was withdrawn and centrifuged at 500 rpm for 5 min at 4°C, and the supernatant was discarded. Cells were collected, resuspended in complete RPMI 1640 medium, and incubated for 18 h in cell culture plates. The medium was then exchanged for fresh RPMI 1640, and LPS (200 ng/mL) was added in the presence or absence of RRE for 24 h. All animal studies were performed according to the Guide for the Animal Care and Use Committee of the Korea Institute of Oriental Medicine (reference number: 14-079).

### 2.4. Cell Viability Assay

Cell viability was analyzed by CCK assay according to the manufacturer's instructions. RAW 264.7 cells were plated at a density of 5 × 10^5^ cells/mL in a 96-well plate and treated with varying concentrations of RRE for 24 h. CCK solutions were added to each well, and the cells were further incubated for 1 h. The absorbance was measured at 450 nm using an ELISA reader (Infinite M200, Tecan, Männedorf, Switzerland).

### 2.5. Measurement of NO Levels

RAW 264.7 cells were plated at 5 × 10^5^ cells/mL in 96-well plates and pretreated with RRE for 1 h prior to stimulation with LPS (200 ng/mL) for 24 h. Nitrite levels in culture media were determined using the Griess reagent (1% sulfanilamide, 0.1% naphthylethylenediamine dihydrochloride, and 2.5% phosphoric acid) and incubated at room temperature (RT) for 5 min [22]. The absorbance was then measured at 570 nm using an ELISA reader. The quantity of nitrite in the samples was calculated with sodium nitrite as a standard. All measurements were performed in triplicate and expressed as NO micromolar concentrations.

### 2.6. Determination of Cytokines

The concentrations of TNF-*α*, IL-6, and IL-1*β* in the supernatant were assayed using mouse ELISA antibody kits, according to the manufacturer's instructions. For ELISA, 5 × 10^5^ RAW 264.7 cells/mL were seeded on 24-well culture plates. The cells were pretreated with various concentrations of RRE for 1 h and further challenged with LPS for 24 h at 37°C with 5% CO_2_. The serum was centrifuged at 13,000 rpm for 10 min, following which the supernatant and serum were collected. The cytokine concentrations were measured from a standard curve developed using a known concentration of recombinant TNF-*α*, IL-6, and IL-1*β*.

### 2.7. Preparation of Whole Cell, Cytosolic, and Nuclear Extracts

RAW 264.7 cells were seeded at 1 × 10^6^ cells/mL on six-well plates and were treated with RRE and stimulated with LPS for indicated periods. After incubation, cells were harvested by centrifugation and washed twice with ice-cold PBS. To obtain whole cell lysates, pellets were resuspended in radioimmunoprecipitation assay (RIPA) lysis buffer (Millipore, Bedford, MA, USA) containing protease and phosphatase inhibitors. Cell debris was removed after centrifugation. The cytosolic and nuclear proteins were extracted using NE-PER cytoplasmic and nuclear extraction reagents according to the manufacturer's instructions (Thermo Scientific, Rockford, IL, USA).

### 2.8. Western Blot Analysis

Total protein of whole cell, cytoplasmic, and nuclear extracts was determined using Bradford reagent (Bio-Rad, Hercules, CA, USA). Equal quantities of protein were subjected to sodium dodecyl sulfate-polyacrylamide gel electrophoresis (SDS-PAGE). The protein was transferred onto a nitrocellulose membrane with a glycine transfer buffer (192 mM glycine, 25 mM Tris-HCl (pH 8.8), and 20% MeOH (v/v)). The membranes were blocked and incubated with specific primary antibody at 4°C overnight followed by incubation with the horseradish peroxidase- (HRP-) linked secondary antibody at RT for 1 h. The signals were detected using SuperSignal West Femto Chemiluminescent Substrate (Thermo Scientific, Rockford, IL, USA). Protein levels were quantified using a Davinch-Chemi*™* Chemiluminescence Imaging System CAS-400SM (Core Bio, Seoul, Korea).

### 2.9. RNA Isolation and Real-Time Reverse Transcription-Polymerase Chain Reaction (Real-Time RT-PCR)

Total RNA was extracted from RAW 264.7 cells using the easy-BLUE*™* RNA extraction kit (iNtRON Biotech, Daejeon, Korea) in accordance with the manufacturer's instructions. Specifically, 1 *μ*g of total RNA was used for the first strand cDNA synthesis using AccuPower® CycleScript RT PreMix (Bioneer, Daejeon, Korea). The oligonucleotide primers for real-time RT-PCR used with mouse macrophage cDNA are listed in Table 1. The reactions were performed in triplicate in a 20 *μ*L total volume: 0.3 *μ*M final concentrations of each primer, 10 *μ*L of AccuPower® 2x GreenStar qPCR Master Mix (Bioneer, Daejeon, Korea), and 2 *μ*L of template DNA. The following PCR conditions were applied for TNF-*α*, IL-6, IL-1*β*, iNOS, COX-2, HO-1, and *β*-actin: 40 cycles at 94°C for 15 s and 60°C for 1 min [23]. Amplification and analyses were performed with a QuantStudio 6 Flex Real-Time PCR System (Thermo Scientific, Rockford, IL, USA). Samples were compared using the relative CT method [23]. The fold increase or decrease in gene expression was determined relative to a blank control after normalization to the *β*-actin gene using 2^−ΔΔC^T.

### 2.10. Chromatographic Conditions

Chromatographic analysis was conducted following a modified version of the method described previously [24–26]. For standardization of RRE, chromatographic analysis was performed using a Hitachi HPLC-DAD system (Hitachi Co., Tokyo, Japan) consisting of a pump (L-2130), autosampler (L-2200), column oven (L-2350), and diode array UV/VIS detector (L-2455). An OptimaPak C18 column (5 *μ*m, 4.6 × 250 mm, RS Tech, Daejeon, Korea) was maintained at 40°C for sample analysis. Output signals from the detector were recorded using EZChrom Elite software from Hitachi. The fingerprinting was conducted with the chemical standards rhapontisterone B, pomolic acid, and echinoynethiophene A (ChemFaces, Wuhan, Hubei, China). The standard solutions were prepared by dissolving each marker component in 100% methanol at 1 mg/mL. RR powder was weighed and dissolved in methanol at 5 mg/mL for analysis. The standard solutions and sample were injected at a volume of 10 *μ*L and were detected at a UV wavelength of 254 nm. The mobile phase was water with 0.1% acetic acid (A) and acetonitrile with 0.1% acetic acid (B) at a flow rate of 1.0 mL/min with the following gradient flow: 10% B at 0–10 min, 10–50% B at 10–50 min, 50% B at 50–55 min, and 50–100% B at 55–60 min. The identification of the peaks was based on the UV spectrum and retention time of each marker component from the RRE.

### 2.11. Statistical Analysis

Results are expressed as the mean ± standard deviation (SD) for all experiments, and all quantitative data are representative of at least three independent experiments. Student's* t-*tests were used to determine the statistical significance of differences between each treated group and the negative control (LPS group). Values of ^*∗*^ *p* < 0.01 and ^*∗∗*^ *p* < 0.001 were considered to be statistically significant.

## 3. Results

### 3.1. Effects of RRE on Cell Viability

**Figure 1:**
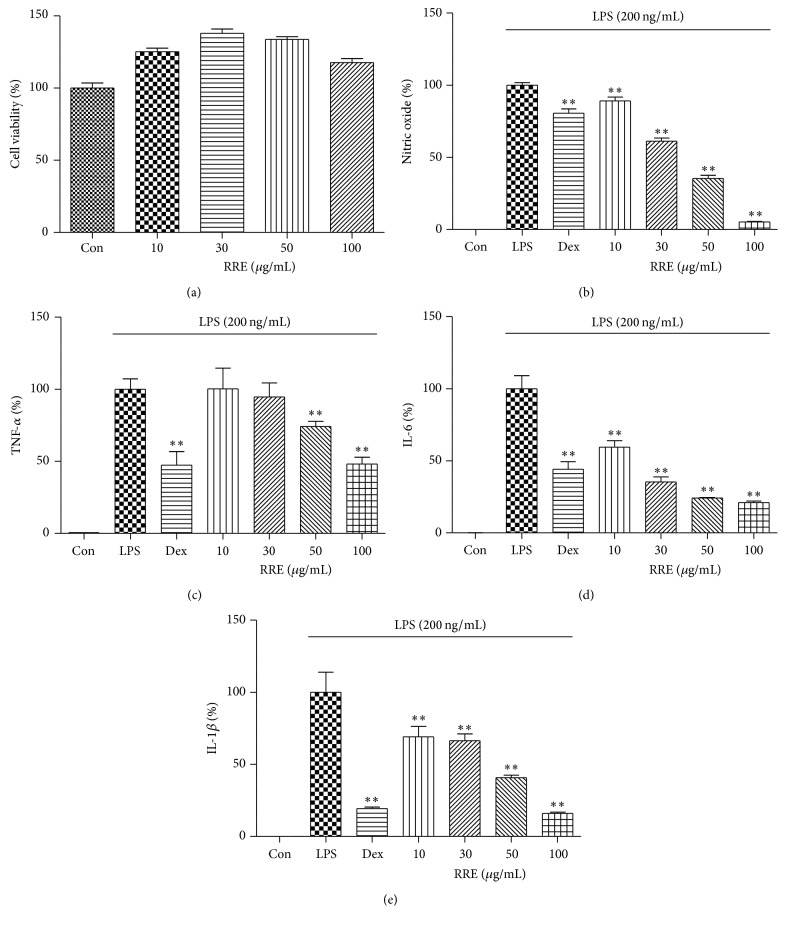
Effects of RRE on (a) cell viability and (b–e) production of inflammatory mediators in RAW 264.7 cells. Cells were pretreated with RRE for 1 h prior to 24 h incubation with LPS. (a) Cell viability was measured by CCK assay. (b) NO content in the conditioned media was determined using Griess reagent. Levels of (c) TNF-*α*, (d) IL-6, and (e) IL-1*β* in the media were measured by ELISA. As a control, cells were incubated with vehicle alone. Data represent the mean ± SD of duplicate determinations from three independent experiments. ^*∗∗*^ *p* < 0.001 was calculated from comparing with LPS-stimulation value.

The cytotoxicity of RRE on RAW 264.7 cells was evaluated by CCK assay after 24 h of treatment. The viability of the cells treated with RRE is shown in Figure 1(a), which indicates that a concentration of up to 100 *μ*g/mL RRE was associated with no significant change in overall cell viability. Subsequent experiments were therefore performed with concentrations up to 100 *μ*g/mL.

### 3.2. Inhibitory Effects of Treatment with RRE on NO Production

Because NO production has been correlated with various inflammatory diseases, we decided to investigate the suppressive effects of RRE treatment on the NO level in macrophages stimulated with LPS. The supernatant was treated with a range of RRE concentrations (10–100 *μ*g/mL) for 1 h followed by stimulation with LPS for 24 h, and NO production was measured using Griess reagent. We found that RRE dramatically inhibited the release of NO in a dose-dependent manner upon LPS stimulation (Figure 1(b)).

### 3.3. Effects of RRE Treatment on Inflammatory Cytokine Secretion and mRNA Expression

**Figure 2:**
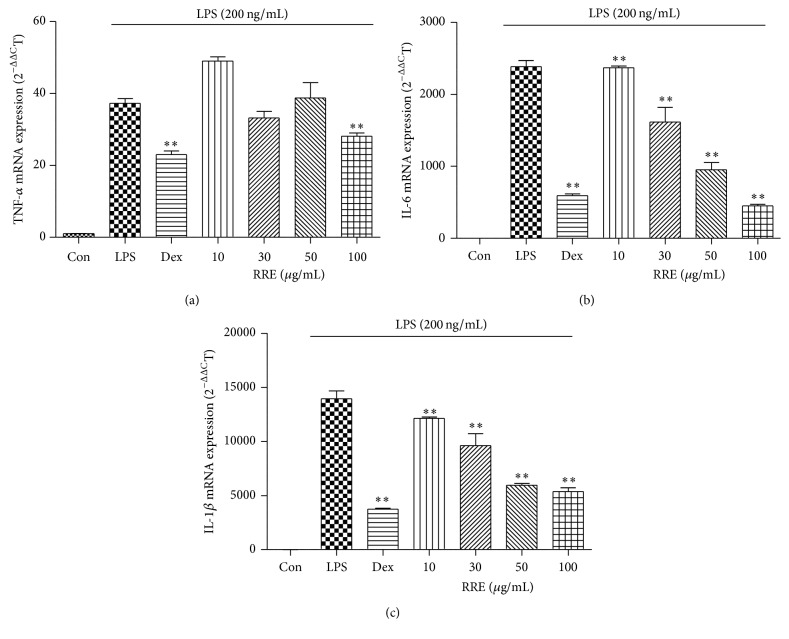
Effects of RRE treatment on LPS-induced (a) TNF-*α*, (b) IL-6, and (c) IL-1*β* mRNA expression in RAW 264.7 cells. Cells were pretreated with RRE for 1 h and stimulated with LPS for an additional 6 h. mRNA levels were measured by real-time RT-PCR. Data represent the mean ± SD of duplicate determinations from three independent experiments. ^*∗∗*^ *p* < 0.001 was calculated from comparing with LPS-stimulation value.

ELISA and real-time RT-PCR were used to evaluate cytokine secretion and mRNA expression of TNF-*α*, IL-6, and IL-1*β* in macrophages following treatment with RRE. As shown in Figures 1(c)–1(e), the secretion of three inflammatory cytokines was significantly inhibited by treatment with RRE in a dose-dependent manner. Notably, treatment with the highest concentration (100 *μ*g/mL) of RRE strongly suppressed secretion more than did the positive control (10 *μ*M dexamethasone). Consistent with this, mRNA expression of IL-6 and IL-1*β* was inhibited by RRE treatment in a dose-dependent fashion, and this decrease was statistically significant (Figures 2(b) and 2(c)). RRE treatment, however, had no effect on TNF-*α* mRNA expression except at the highest concentration (100 *μ*g/mL) (Figure 2(a)).

### 3.4. Effects of RRE on iNOS, COX-2, and HO-1 Expression Levels

**Figure 3:**
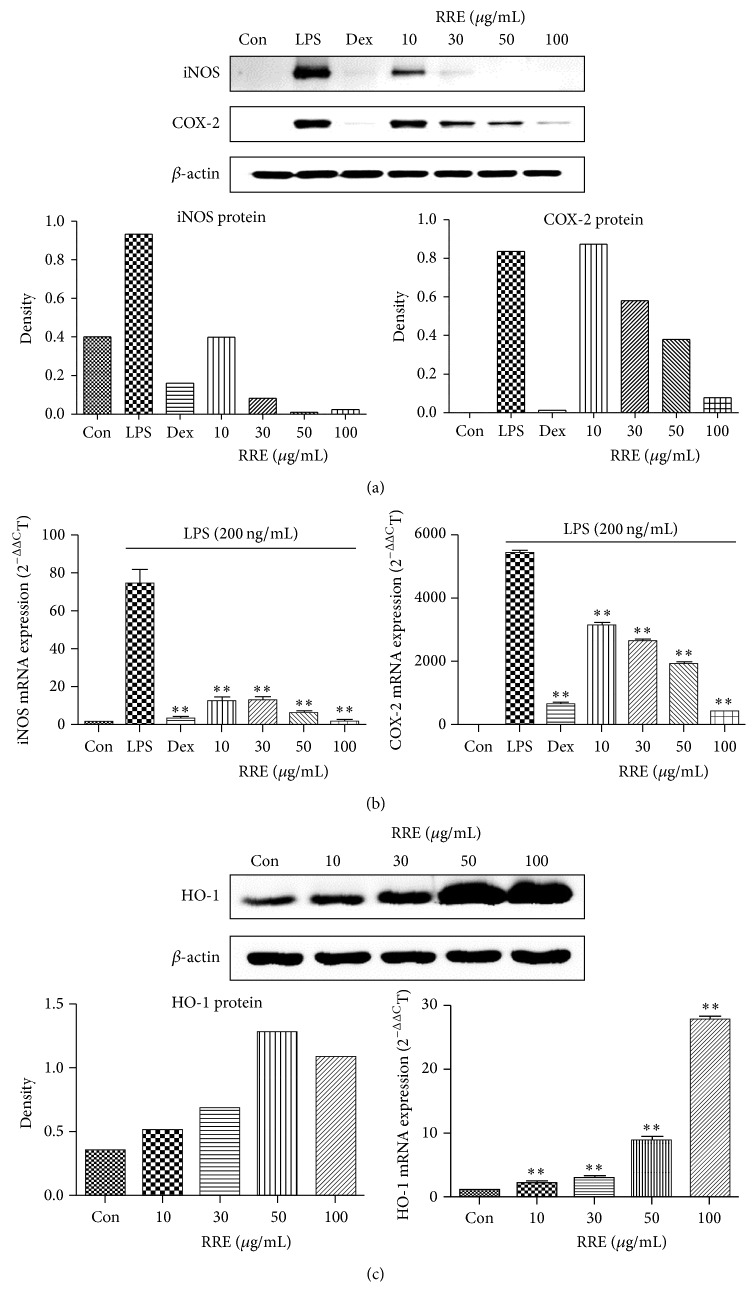
Effects of RRE treatment on the expression of (a) cellular proteins iNOS and COX-2, (b) their mRNAs, and (c) HO-1 in RAW 264.7 cells. Cells were treated with (a, b) LPS alone or with LPS and RRE for 24 h and (c) RRE alone for 6 h. *β*-actin served as a control. Results are representative of three independent experiments. ^*∗∗*^ *p* < 0.001 was calculated from comparing with LPS-stimulation value.

The effects of RRE treatment on protein and mRNA expression of iNOS, COX-2, and HO-1 in RAW 264.7 cells were investigated by Western blot and real-time RT-PCR analysis. As shown in Figure 3(a), RRE treatment significantly decreased the protein levels of iNOS in a concentration-dependent manner. Protein levels of COX-2 were also greatly reduced at concentrations of 30–100 *μ*g/mL. Moreover, as shown in Figure 3(c), HO-1 protein expression was dramatically and dose dependently increased by RRE treatment. According to the real-time RT-PCR analysis results, treatment with RRE markedly repressed the iNOS and COX-2 mRNA levels and induced the expression of HO-1 mRNA (Figures 3(b) and 3(c)).

### 3.5. Effects of RRE Treatment on LPS-Induced Nuclear Translocation of NF-*κ*B and the Phosphorylation of I*κ*B*α*

**Figure 4:**
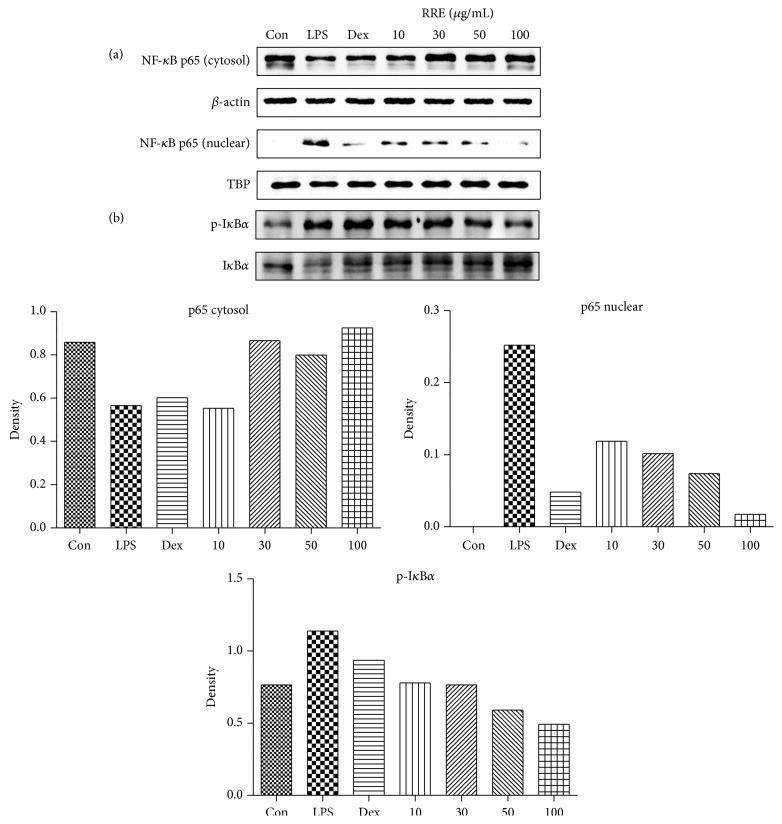
Effects of RRE treatment on (a) NF-*κ*B p65 translocation into the nucleus and (b) phosphorylation of I*κ*B*α* in RAW 264.7 cells. Cells were pretreated with RRE for 1 h and stimulated with LPS for an additional (a) 1 h or (b) 30 min. *β*-actin and TATA box-binding protein (TBP) were used for cytosolic and nuclear control proteins, respectively. Results are representative of three independent experiments.

Because they are important regulators of the inflammatory response, we also examined the effects of RRE treatment on LPS-induced changes in the levels of NF-*κ*B, phospho-I*κ*B*α*, and I*κ*B*α*. RRE treatment strongly inhibited LPS-induced translocation of p65 to the nucleus at a dose of 10 *μ*g/mL or greater (Figure 4(a)). TATA box-binding protein (TBP) was used as an internal control. In addition, RRE treatment markedly suppressed cytoplasmic I*κ*B*α* degradation and phosphorylation in a dose-dependent manner (Figure 4(b)). These results suggested that treatment with RRE effectively blocks LPS-induced activation of NF-*κ*B, as well as that of I*κ*B*α*, in macrophages.

### 3.6. Effects of RRE Treatment on MAPKs Phosphorylation in LPS-Stimulated RAW 264.7 Cells

**Figure 5:**
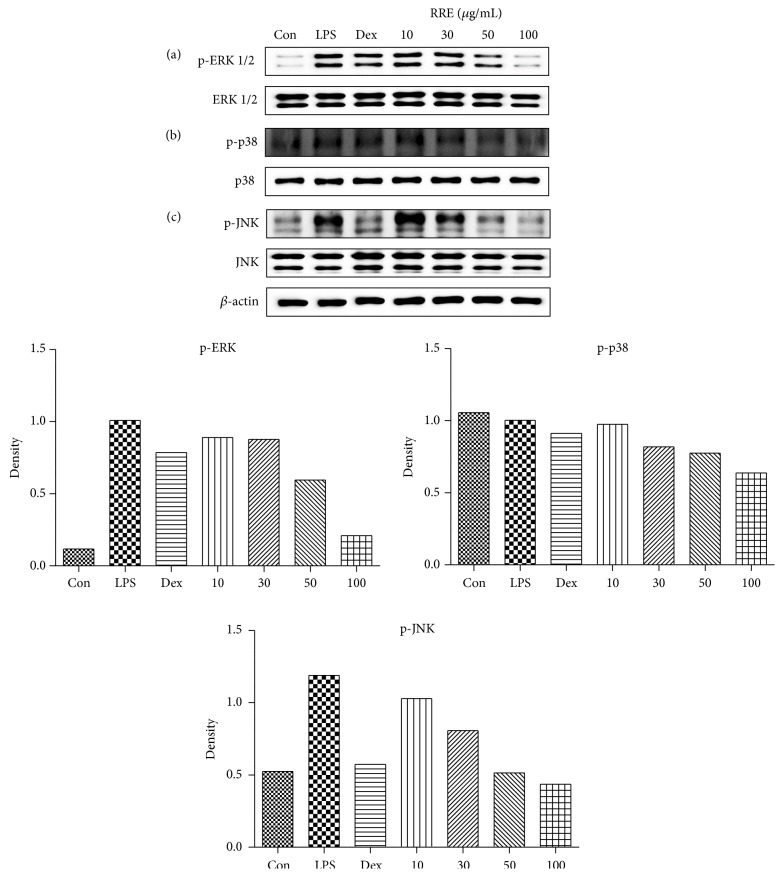
Effects of RRE treatment on the phosphorylation of (a) ERK, (b) p38, and (c) JNK MAPKs in RAW 264.7 cells. Cells were treated with RRE for 1 h and challenged with LPS for 30 min. ERK, p38, and JNK, as well as their phosphorylated forms (p-ERK, p-p38, and p-JNK), were examined by Western blot. Results are representative of three independent experiments.

Because the three MAPKs, ERK1/2, p38, and JNK, are essential regulators of the inflammatory response [27], we examined the effects of RRE treatment on the activation of MAPKs by Western blot analysis using specific antibodies to test for their phosphorylated forms. As displayed in Figure 5, RRE treatment markedly inhibited the phosphorylation of ERK, p38, and JNK in a dose-dependent manner.

### 3.7. Effects of RRE Treatment on Inflammatory Cytokine Production in LPS-Induced Mouse Peritoneal Macrophages

**Figure 6:**
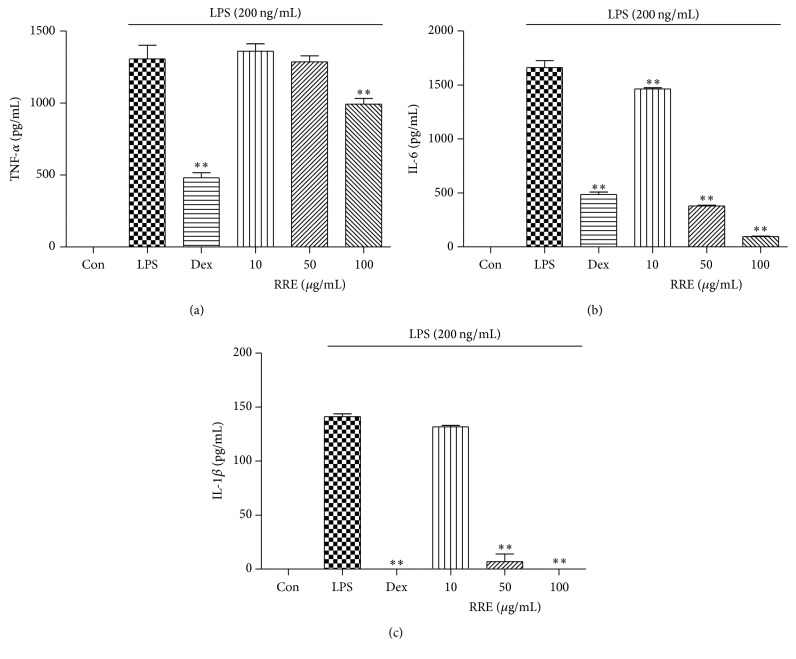
Effect of RRE treatment on the production of (a) TNF-*α*, (b) IL-6, and (c) IL-1*β* cytokines in LPS-stimulated mouse peritoneal macrophages. Cells were treated with RRE for 1 h and stimulated with LPS for 24 h. Cytokine production was measured by ELISA. Data represent the mean ± SD of duplicate determinations from three independent experiments. ^*∗∗*^ *p* < 0.001 was calculated from comparing with LPS-stimulation value.

After examining the anti-inflammatory effect of RRE treatment on LPS-stimulated RAW 264.7 cells, we wanted to confirm the inhibitory effect of RRE treatment in mouse primary macrophages. We measured the levels of three inflammatory cytokines, including TNF-*α*, IL-6, and IL-1*β*. As shown in Figure 6(a), RRE treatment at a concentration of 100 *μ*g/mL only slightly repressed TNF-*α* secretion. However, RRE treatment significantly inhibited secretion of IL-6 and IL-1*β* in a dose-dependent manner (Figures 6(b) and 6(c)). These results indicate that treatment with RRE effectively inhibits the inflammatory response in primary macrophages.

### 3.8. Representative Chromatograms of the Constituents in RRE

**Figure 7:**
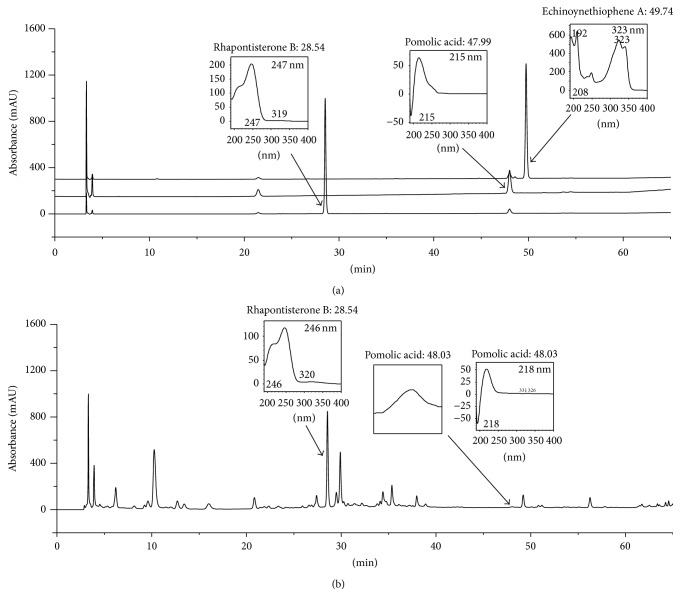
HPLC-DAD analysis of the constituents in RR. Rhapontisterone B, pomolic acid, and echinoynethiophene A in (a) standard mixture and (b) RRE were identified at 254 nm.

The constituents of RR were determined by HPLC analysis, and each peak of UV spectra was compared with that of representative standard compounds. At 254 nm, HPLC-DAD analysis revealed that the compounds found in RRE showed the following approximately coincident UV spectra and retention times: rhapontisterone B (246 nm, *t*_R_: 28.54 min) and pomolic acid (218 nm, *t*_R_: 48.03 min) compared with those of standard compounds such as rhapontisterone (247 nm, *t*_R_: 28.54 min) and pomolic acid (215 nm, *t*_R_: 47.99 min) (Figure 7). Echinoynethiophene was not detected in RRE in this study.

## 4. Discussion

RR is an important traditional medicinal herb and is generally used in East Asia to treat symptoms associated with inflammatory diseases. A previous study reported the erythrocyte immune function of RRE in rats [28]. The molecular mechanism underlying the effect of RRE on LPS-induced inflammation is still unknown. In the present study, we investigated the anti-inflammatory activity and its potential mechanism of RRE in LPS-stimulated RAW 264.7 cells and mouse primary macrophages in an* in vitro* inflammation model. To evaluate the anti-inflammatory effects of RRE, we used several* in vitro* assays including the inhibition of nitrite, iNOS, COX-2 production, and proinflammatory cytokine secretion (or mRNA expression). To explore the potential mechanisms of anti-inflammatory activity, we also evaluated the induction of HO-1, the phosphorylation of MAPK, and the activation of the NF-*κ*B signaling pathway in LPS-stimulated macrophages without appreciable cytotoxic effects. Therefore, the objective of our study is to elucidate the potential anti-inflammatory effects and the mechanisms of RRE within pharmacologically noncytotoxic concentration levels.

First, we determined the cytotoxicity in RAW 264.7 cells by treatment with RRE because RRE cytotoxicity must be evaluated before therapeutic applications can be considered. We found that treatment with RRE did not affect cell viability at concentrations below 100 *μ*g/mL. To exclude potential cytotoxicity, the experiments conducted in this study were limited to a concentration of 100 *μ*g/mL RRE. Using this information, we examined the production of several inflammatory mediators. Production of NO is mediated primarily by NOSs, and prostaglandin E_2_ (PGE_2_) is generated by COX-2, and both play essential roles in inflammation and immune regulation [29]. Thus, we determined the inhibitory effects of RRE treatment on NO, iNOS, and COX-2 production induced by LPS stimulation. We found that RRE treatment downregulated NO secretion and inhibited iNOS, COX-2 protein, and mRNA expression in a concentration-dependent manner. RRE treatment also very strongly reduced iNOS expression, at both the protein and mRNA levels, at all concentrations. Based on these results, we suggest that the inhibitory effect of RRE on the inhibition of iNOS expression correlates with the repression of NO secretion. We conclude that treatment with RRE more effectively suppresses NO and iNOS production compared with other inflammatory parameters.

The production of NO and iNOS is associated with the induction of HO-1 [30]. In addition, recent pharmacological studies have investigated HO-1 signal transduction to understand its anti-inflammatory action [31]. Thus, we investigated the effect of RRE treatment on the mRNA and protein levels of HO-1 in unstimulated cells. In our experiments, treatment with RRE significantly induced HO-1 expression in a dose-dependent manner in macrophage RAW 264.7 cells. In particular, we found that 100 *μ*g/mL RRE strongly induced HO-1 expression at both the mRNA and the protein levels. These data suggest that the anti-inflammatory activity of RRE may operate through HO-1 induction.

We also found that treatment with RRE repressed the production of inflammatory cytokines and their mRNA expression level in macrophages. In response to LPS, macrophages release a number of proinflammatory cytokines, including TNF-*α*, IL-6, and IL-1*β*. In this study, treatment with RRE slightly inhibited TNF-*α* production at higher concentrations and more strongly suppressed IL-6 and IL-1*β* production at all doses.

To further clarify the anti-inflammatory property of RRE and its inhibitory effect on inflammatory mediators, we evaluated the effects of RRE treatment on the activation of two signaling pathways, NF-*κ*B and MAPKs, in LPS-stimulated macrophages. It is known that NF-*κ*B and MAPK are important signal pathways, and these proteins are key regulators of a number of genes involved in the immune and inflammatory response [32]. Activation of the NF-*κ*B pathway leads to I*κ*B*α* phosphorylation and degradation, promoting the translocation of the NF-*κ*B subunit p65 to the nucleus to initiate transcription of inflammatory cytokines and trigger the activation of inflammation-related enzymes [13–15]. Given this, we decided to investigate p65 levels in cytoplasmic and nuclear extracts as well as the phosphorylation status of I*κ*B*α*. Western blot analysis showed that treatment with RRE reduced NF-*κ*B p65 translocation to the nucleus through inhibition of I*κ*B*α* phosphorylation and degradation in a dose-dependent manner. Additionally, we investigated the MAPK pathway, which includes ERK, p38, and JNK. We found that treatment with RRE significantly reduced the phosphorylation of ERK and JNK in a dose-dependent manner, suggesting that the activation of MAPK signaling is inhibited by RRE treatment. We suggest that the anti-inflammatory activity of RRE may be explained by the decrease in MAPK signaling and inhibition of NF-*κ*B.

We further investigated the suppressive effect of RRE treatment on proinflammatory cytokine production in LPS-stimulated mouse primary macrophages to confirm its anti-inflammatory activity. Treatment with RRE significantly inhibited the production of IL-6 and IL-1*β* cytokines in primary macrophages but did not suppress TNF-*α* cytokine production. These results implied that treatment with RRE represses the inflammatory response by inhibiting the production of proinflammatory cytokines in both the murine cell line and primary cells.

In Figure 7, we confirmed two main components (rhapontisterone B and pomolic acid) of RRE, consistent with previously published results [24, 25]. One study reported that pomolic acid exerts anti-inflammatory and apoptotic activities by reduced carrageenan-induced paw edema in mice and in human polymorphonuclear (PMN) cells, respectively [33]. This suggested that the anti-inflammatory activity of RRE might be related to the active components of RRE, such as pomolic acid. However, additional studies will be required to fully understand the main constituents of RR and their biological activities.

In conclusion, the results of this study provide evidence that treatment with RRE inhibits the production of inflammatory mediators, including NO, inflammatory cytokines, iNOS, and COX-2, in LPS-stimulated RAW 264.7 cells. These effects can be attributed to the inhibition of NF-*κ*B activation by the phosphorylation and degradation of I*κ*B*α* and the blockade of MAPK phosphorylation. The induction of HO-1 expression by RRE treatment affects the repression of inflammatory mediators. In addition, treatment with RRE suppressed the production of proinflammatory cytokines in mouse primary macrophages. Also, two main components of RRE including rhapontisterone B and pomolic acid might be closely related to anti-inflammatory effects of RRE. These results suggest that RRE has potential value as candidate for inflammatory therapeutic phytomedicine.

## Figures and Tables

**Table 1:** Primers used for real-time RT-PCR.

Target gene	Primer sequence
TNF-*α*	F: 5′-TTCTGTCTACTGAACTTCGGGGTGATCGGTCC-3′
	R: 5′-GTATGAGATAGCAAATCGGCTGACGGTGTGGG-3′

IL-6	F: 5′-TCCAGTTGCCTTCTTGGGAC-3′
	R: 5′-GTGTAATTAAGCCTCCGACTTG-3′

IL-1*β*	F: 5′-ATGGCAACTGTTCCTGAACTCAACT-3′
	R: 5′-CAGGACAGGTATAGATTCTTTCCTTT-3′

iNOS	F: 5′-GGCAGCCTGTGAGACCTTTG-3′
	R: 5′-GCATTGGAAGTGAAGCGTTTC-3′

COX-2	F: 5′-TGAGTACCGCAAACGCTTCTC-3′
	R: 5′-TGGACGAGGTTTTTCCACCAG-3′

HO-1	F: 5′-TGAAGGAGGCCACCAAGGAGG-3′
	R: 5′-AGAGGTCACCCAGGTAGCGGG-3′

*β*-actin	F: 5′-AGAGGGAAATCGTGCGTGAC-3′
	R: 5′-CAATAGTGATGACCTGGCCGT-3′

F: forward; R: reverse.
